# Direct photo-patterning on anthracene containing polymer for guiding stem cell adhesion

**DOI:** 10.1186/s40824-016-0072-4

**Published:** 2016-08-03

**Authors:** Jungmok You, June Seok Heo, Hyun Ok Kim, Eunkyoumg Kim

**Affiliations:** 1 Department of Plant & Environmental New Resources, Kyung Hee University, 1732 Deogyeong-daero, Giheung-gu, Yongin-si, Gyeonggi-do 446-701 South Korea; 2 Cell Therapy Center, Severance Hospital, Yonsei University College of Medicine, Seoul, South Korea; 3 Department of Laboratory Medicine, Yonsei University College of Medicine, Seoul, South Korea; 4 Department of Chemical and Biomolecular Engineering, Yonsei University, 262 Seongsanno, Seodaemun-gu, Seoul 120-749 South Korea

**Keywords:** Anthracene, Fluorescent polymer, Photolithography, Polymer pattern film, Photoreaction, Human mesenchymal stem, Cell patterning

## Abstract

**Background:**

Various micropatterned surfaces capable of guiding the selective adhesion of biomolecules such as proteins and cells are of great interests in biosensor, diagnostics, drug screening, and tissue engineering. In this study, we described a simple photo-patterning method to prepare micro-patterned films for stem cell patterning using anthracene containing polymers (PMAn). This micro patterned polymer film was prepared by the facile photo-reaction of anthracene units in polymer backbone structure.

**Results:**

The UV irradiation of PMAn through a photomask resulted in the quenching of fluorescent intensity as well as the changes in surface wettability from hydrophobic to hydrophilic surface. As a result, UV exposed regions of PMAn film show lower fluorescent intensity as well as higher proliferation rate of mesenchymal stem cells (MSCs) than unexposed region of PMAn film. Furthermore, the selective MSC attachment was clearly observed in the UV exposed regions of PMAn film.

**Conclusion:**

We developed a simple cell patterning method with a fluorescent, biocompatible, and patternable polymer film containing anthracene units. This method provides a facile stem cell patterning method and could be extended to various patterning of biomaterials without labor-intensive preparation and no pre-treatment for complex interactions of cell-microenvironment.

## Background

Micropatterned surfaces that can selectively interact with biomaterials such as proteins and cells are of great interest in genomics, diagnostics, drug screening, and tissue engineering, as these “micro-scale patterned surfaces” enable us to define the adhesion of single cells or cell groups [1–3]. Beyond the control of cell adhesion, these surfaces are particularly valuable for designing and developing cell culture systems which reflect better the complexity of cell-microenvironment interactions in order to regulate cell phenotype and cell fate [4–6].

A number of micropatterning approaches have been extensively exploited including microcontact printing, photoresist lithography, microfluidics, and self-assembled monolayers (SAMs) [7–11]. Also, a number of laboratories, including our own, have been performing surface engineering with poly (ethylene glycol) (PEG) photolithography to control cell-surface interactions, originating from anti-fouling effects of PEG hydrogel [12–14]. However, SAM method with alkylthiols requires labor intensive preparation and also is only applicable to gold and glass substrate. Also, the major limitations of PEG hydrogel micropatterned surfaces is the instability of PEG adhesion on substrates. The stability of PEG hydrogel micropatterns is influenced by not only the molecular weight and concentration of PEG but also the treatment of silane as a coupling layer. In general, thiol-silane anchored PEG hydrogel are durable for up to 3 days but this hydrogel started being detached after 4 days, in spite of the anchoring of hydrogel microstructures with silane coupling agents [15]. Thus, simple but solid methods for cell micropatterns still remains a significant challenge.

The patterning of functional polymers with conductive and fluorescent properties has recently garnered considerable attention for sensor, bioengineering, display and electrical circuits because of their specific opto-electronic properties [16–19]. Recently, we reported direct photopatterning approaches for functional polymers to avoid decomposition of the functional group in polymer structure during the patterning step [20–22]. Leveraging simple and direct photo-patterning process, we have developed a highly fluorescent and biocompatible *p*-phenylene vinylene (PPV) polymer pattern film that allows stem cell micropatterns [23]. This enables us to easily detect the location of cells or other biomaterials without the labeling cells because the pattern is fluorescent.

For the application of direct photo-patterning, it is important to prepare a patternable fluorescent polymer. Anthracnee moleuclue has been frequently utilized to fabriciate photo-responsive biocompatible drug delivery systems due to the its ability of photo-truggered reaction upon UV exposure [24, 25]. In this study, we report a photo patternable anthracene containing fluorescent polymer (PMAn) for the selective patterning of mesenchymal stem cells (MSCs). We utilized micro polymer (PMAn) patterns formed by direct photo-reaction of anthracene units for guiding stem cell adhesion.

## Methods

### Materials

Methylene bridged anthracene polymer (PMAn) was synthesized via Friedel-Crafts alkylation reaction [26, 27]. Weight average molecular weight (*M*_w_) of the resultant polymer by gel permeation chromatography (GPC) was 6700 with *M*_*w*_*/M*_*n*_ of 1.7. α-Minimum Essential Medium (α-MEM), Fetal Bovine Serum (FBS), antibiotics (Penicillin/Streptomycin), Phosphate-Buffered Saline (PBS, pH 7.4), Trypsin/EDTA (0.05 %), and Tryphan blue (0.4 %) were purchased from Gibco (Invitrogen, USA). Albumin (5 %) was purchased from Green cross corporation (Korea). Bone Marrow (BM)-derived Mesenchymal Stem Cells (MSCs), on patient compliance, were used for this study. A frozen stock of MSCs was provided by Cell Therapy Center, Severance hospital (from University of Yonsei, Seoul, Korea) at passage 3. Other chemicals and solvents were purchased from Aldrich.

### Instruments

^1^H spectrum was determined on a Bruker ARX-300 spectrometer. The average molecular weight of the polymer was characterized by a gel permeation chromatography (GPC) (model: Waters R-401 ALC/GPC) with THF as an eluent and polystyrene standard for calibration. Fluorescence spectra were obtained with a luminescence spectrometer (PerkinElmer, Model LS55) under excitation at 370 nm. The polymer films were illuminated with a UV lamp (Rolence Enterprise, Inc., Taiwan, power: 13.05 mW/cm^2^), model POWERARC UV 100. The surface wettability was investigated by a water (DI) drop contact angle measurement using Contact Angle Meter-CAM 101 model (KSV Instruments Ltd, FINLAND). AFM analysis was carried out in room temperature with a Dimension 3100 SPM equipped with Nanoscope IVa devised by Digital Instruments from Santa Barbara, CA. The fluorescent patterns such as were imaged under Olympus-BX51 fluorescence microscope with WB – dichroic mirror DM500, excitation filter BP450-480 and barrier filter BA515. The optical MSCs patterns were obtained from Olympus inverted research microscope model IX71. To detect cell patterns more in detail, MSCs were observed with field emission-scanning electron microscope (HITACHI S-800, Tokyo, Japan) and the picture was taken by scanning microscope image analysis system (ESCAN-4000, Bummi Universe, Tokyo, Japan)

### The preparation of PMAn film and PMAn patterned substrates

PMAn films having average thickness of 185 nm were prepared by spin coating of chloroform solution of polymers (1 wt %) at 1200 rpm for 15 s and then dried under room temperature for solvent removal. These pristine PMAn films, without pattern, were used for the spectroscopic measurements, contact angle measurement, and the proliferation assay of MSCs. For fluorescent pattern formation, the PMAn films were illuminated with a high-intensity UV lamp (13.05 mW/cm^2^) through a photomask. These PMAn patterns were used for the AFM and MSCs pattern.

### The analyses of PMAn surface with contact angle and AFM measurements

Surface wettability of the PMAn films through photo-reaction was investigated by water (DI) drop contact angle measurement. PMAn films were illuminated by high intensity UV source for 1, 3, 5, 10 mins. For surface morphology experiments, the polymer films on silicon wafer were illuminated by a high-intensity UV source for 15 min through a 10 μm line pattered photomask. AFM analyses were carried out at room temperature with a Dimension 3100 SPM equipped with Nanoscope IVa devised by Digital Instruments from Santa Barbara, CA. The AFM tip was oscillated at its resonance frequency (75 kHz). Next, the tip was lifted with fixed distance above the sample surface and scanned at that constant height with a voltage applied.

### Cell culture and proliferation assay on PMAn film substrates

MSCs were thawed, placed at a density of about 10,000 cells/cm^2^ in 15 mL medium (α-MEM supplemented with 10 % FBS, 100 U/mL penicillin and 100 μg/mL streptomycin) in a 75 cm flask (Nunc, Denmark), at 37 °C in 5 % humidified CO_2_. Medium was replaced with fresh α-MEM with 10 % FBS, 100 U/mL penicillin and 100 μg/mL streptomycin every 3 or 4 days and cells were grown to 90–95 % of confluence over about 3–7 days. Once the cells reached confluence, they were detached using 0.05 % Trypsin/EDTA and replaced for further expansion. The MSCs in this study were between passage of 4 and 7.

To investigate the proliferation of MSCs on PMAn film substrates, two PMAn films were prepared on glass substrates by spin coating. One of the films was exposed to UV source for 5 min and the other film was not exposed to UV. The prepared PMAn film substrates were washed with α-MEM supplemented with 10 % FBS, 100 U/mL penicillin and 100 ug/mL streptomycin. Cells (from passage 4) were harvested by treating a solution of 0.05 % Trypsin/EDTA for 3–5 min at 37 °C, placed at 2,000 cells/cm^2^ in unexposed PMAn substrate and UV-exposed PMAn substrate. Cells (from passage 4) were harvested also on a tissue culture polystyrene as a reference experiment. Each well was dispensed with 3 mL α-MEM medium and changed once after 2 days. Cultures were maintained for 5 days and 7 days and then harvested for cell counting, respectively. This proliferation assay was examined at three different times. The growth rates for MSCs on the sample after 5 days and 7 days of culture were determined by counting the number of cells with a hemacytometer after tryphan blue staining in a counting chamber.

### Cell attachment to micro-patterned PMAn substrate

For cell patterning studies, the PMAn pattern on a glass substrate (1.6 cm × 2.8 cm) was prepared by exposing the pristine PMAn film on UV light for 5 min. The micro-patterned substrates were placed in six well plate (Nunc, Denmark) containing culture medium, α-MEM supplemented with 10 % FBS, 100 U/mL penicillin and 100 μg/mL streptomycin. MSCs were detached from the cell culture substrates by trypsinization. The cells at a concentration of 4,000 cells/cm^2^ were seeded on a patterned substrate in six well plate (Nunc, Denmark) and maintained under culture condition for 2 days at 37 °C in 5 % humidified CO_2_.

## Results and discussion

**Fig. 1:**
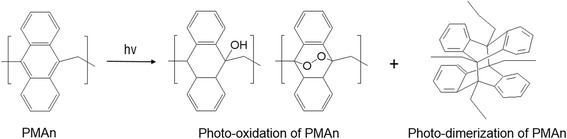
The basic structure and photo-reactions of anthracene containing polymer (PMAn)

**Fig. 2:**
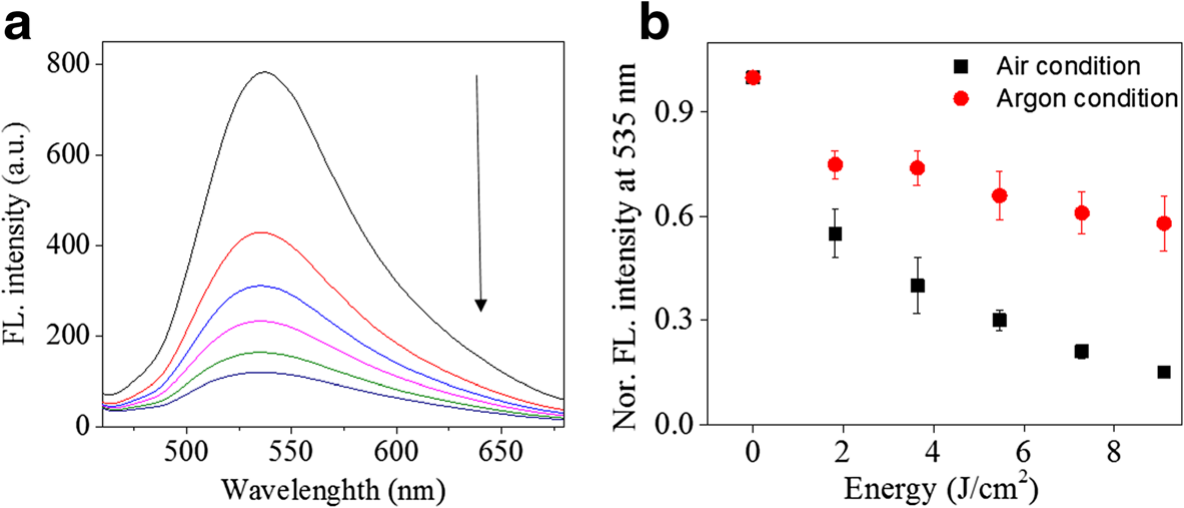
**a** Fluorescence spectra change of a PMAn film depending on UV irradiation time, from top of bottom, 0, 1, 2, 3, 4, 5 min under air. **b** Normalized FL intensity change of the film at 535 nm at different dose under air and argon condition. Film thickness: 185 nm

Figure 1 shows the chemical structure of a highly fluorescent anthracene polymer (PMAn) where a polycyclic aromatic compound, anthracene, is covalently connected through a methylene bridge via Friedel-Crafts alkylation reaction. This polymer is soluble in common organic solvent such as chloroform, tetrahydrofuran, dichloromethane, and acetone. Thus, the polymer film surface was easily prepared with a solution of polymer (1 wt %) in chloroform by spin-coating process. The film thickness of 185 nm was determined by an Alpha-step. The thin film of PMAn showed an emission band maximized at 535 nm (Fig. 2a). After UV exposure for 5 min under air condition, 85 % of the emission intensity was significantly decreased. The fluorescence quenching resulted from the photo-reactions such as photo-oxidation and photo-dimerization of anthracene units exist in polymer backbone structure (Fig. 1) [26, 28–31].

In order to understand the difference in the photo-oxidation and photo-dimerization of PMAn in an atmosphere of air or argon (Ar), the decrease in emission intensity was examined by irradiating the PMAn film at various exposure doses (Fig. 2b).

When UV light was exposed to PMAn film surface in an atmosphere of Ar, the relative emission intensity at 535 nm decreased from 1.0 to 0.58 (42 %). However, in the presence of oxygen, relative emission intensity at 535 nm reduced drastically from 1.0 to 0.15 (85 %). The relative decrease in emission at 535 nm in an atmosphere of Ar was smaller than that in air. This seems to be because the photo-dimerization of the anthracene units in the polymer is the only photochemical reaction in the Ar condition, while the photo-oxidation as well as photo-dimerization took place concomitantly in an atmosphere of air. This result indicated that both photo-oxidation and photo-dimerization led to fluorescence quenching in air.

**Fig. 3:**
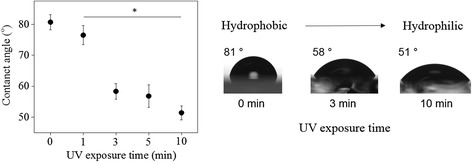
Water contact angle change of a PMAn film depending on UV exposure time under air; p* < 0.05 compared to pristine PMAn film

As shown in Fig. 1, PMAn film undergoes photo-oxidation to produce hydrophilic derivatives including endoperoxide formation, which can cause the change in surface wettability. When PMAn film was exposed to UV source for 0, 1, 3, 5, and 10 mins under air condition, the contact angles of the PMAn film was found to gradually decrease from 81° to 77°, 58°, 56° and 51° (Fig. 3). This result supported that the photo-oxidation of PMAn film resulted in the change in surface wettability to hydrophilic surface, to trigger selective cell adhesion on UV exposed region of PMAn film, as described below.

**Fig. 4:**
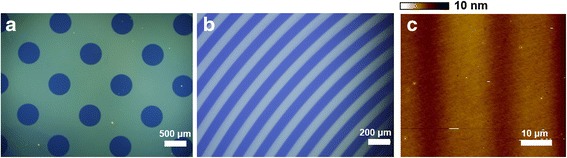
**a** and **b** Fluorescent microscope images of PMAn film with 500 μm diameter circle and 50 μm wide line patterns, respectively, prepared by photo-reactions of PMAn under UV exposure. **c** AFM image of the PMAn film with 10 μm line pattern

The photo-oxidation and photo-dimerization led to fluorescence quenching resulted in fluorescent pattern formation in thin film surface. When PMAn thin film was exposed to UV source through a patterned mask for 5 min, clear fluorescent pattern was formed with 500 μm diameter circle and 50 μm wide line, depending on the shape and size of photomask (Fig. 4a, b).

**Fig. 5:**
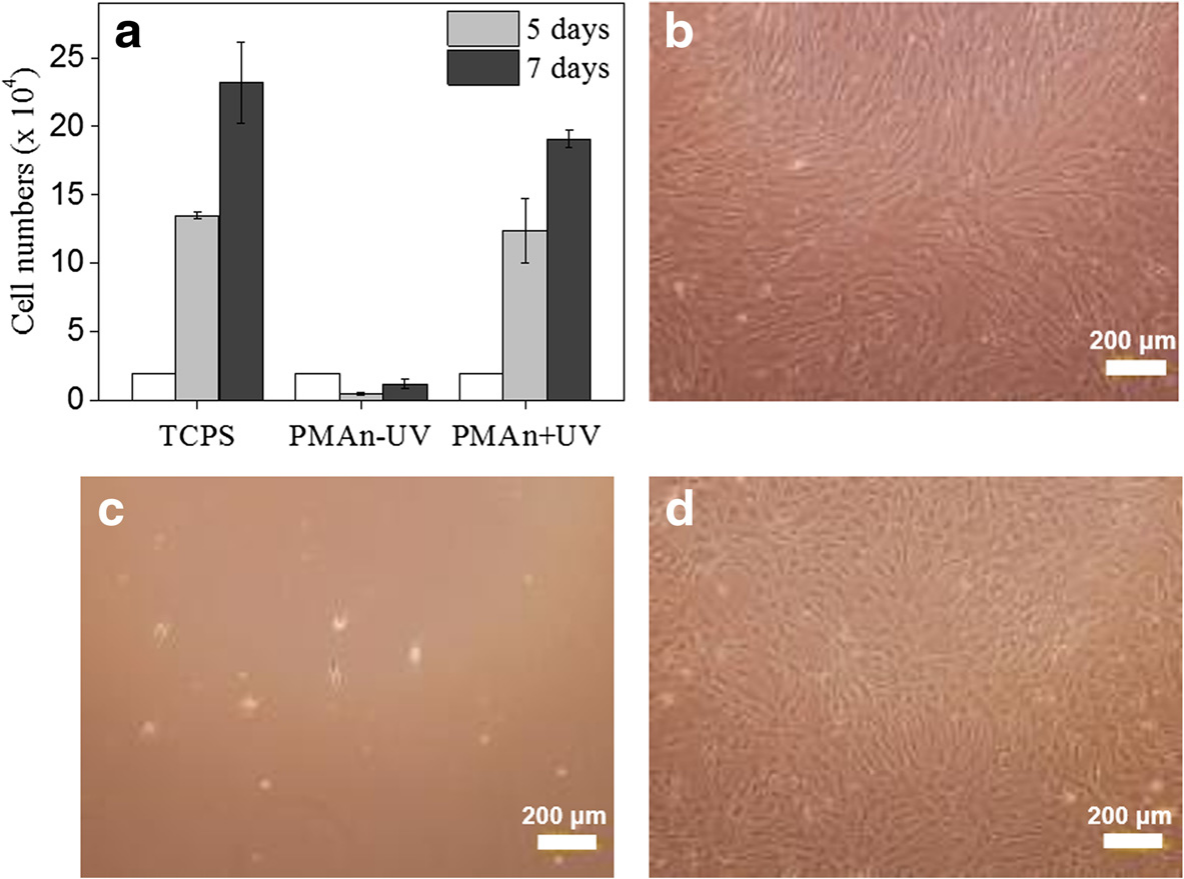
**a** Proliferation rate of MSCs cultured on TCPS as a reference, UV unexposed PMAn film (PMAn-UV), and UV exposed PMAn film (PMAn + UV) in serum contained culture media at day 5 and 7. MSCs were seeded at a density of 2 × 10^3^ cells/cm^2^ at first (white box). **b-d** The optical microscopic images of MSCs cultured on three different substrates, TCPS, PMAn-UV, and PMAn + UV at day 7

Figure 4c shows atomic force microscopy (AFM) image of a 10 μm wide line pattern. The AFM image exhibits that the pattern was formed with an average depth change of 3.3 nm in the exposed region of the film after UV exposure for 15 min. In addition, the AFM analysis exhibits that the surface of the PMAn pattern was very smooth and uniform for both the UV-exposed and unexposed regions, with mean roughness (R_m_) of 1.50. This result indicates that the effect of topography change may be negligible on the selective cell adhesion onto the micro-patterned PMAn film. To investigate the proliferation rate and biocompatibility of MSCs on PMAn film surfaces, cells were cultured on three different substrates: (1) tissue culture polystyrene (TCPS) as a reference, (2) UV unexposed PMAn film (PMAn-UV), and (3) UV exposed PMAn film under air condition (PMAn + UV). As shown in Fig. 5a, the daily proliferation rate of MSCs cultured on PMAn-UV surface was much lower, compared to other substrates. MSCs seeded on PMAn + UV at the density of 2.0 × 10^3^ cells/cm^2^ were increased to 1.8 × 10^4^ cells/cm^2^ (9 times) at day 7, which is comparable to that on TCPS. Figure 5b-d shows the optical microscopic images of MSCs cultured on three different substrates at day 7.

**Fig. 6:**
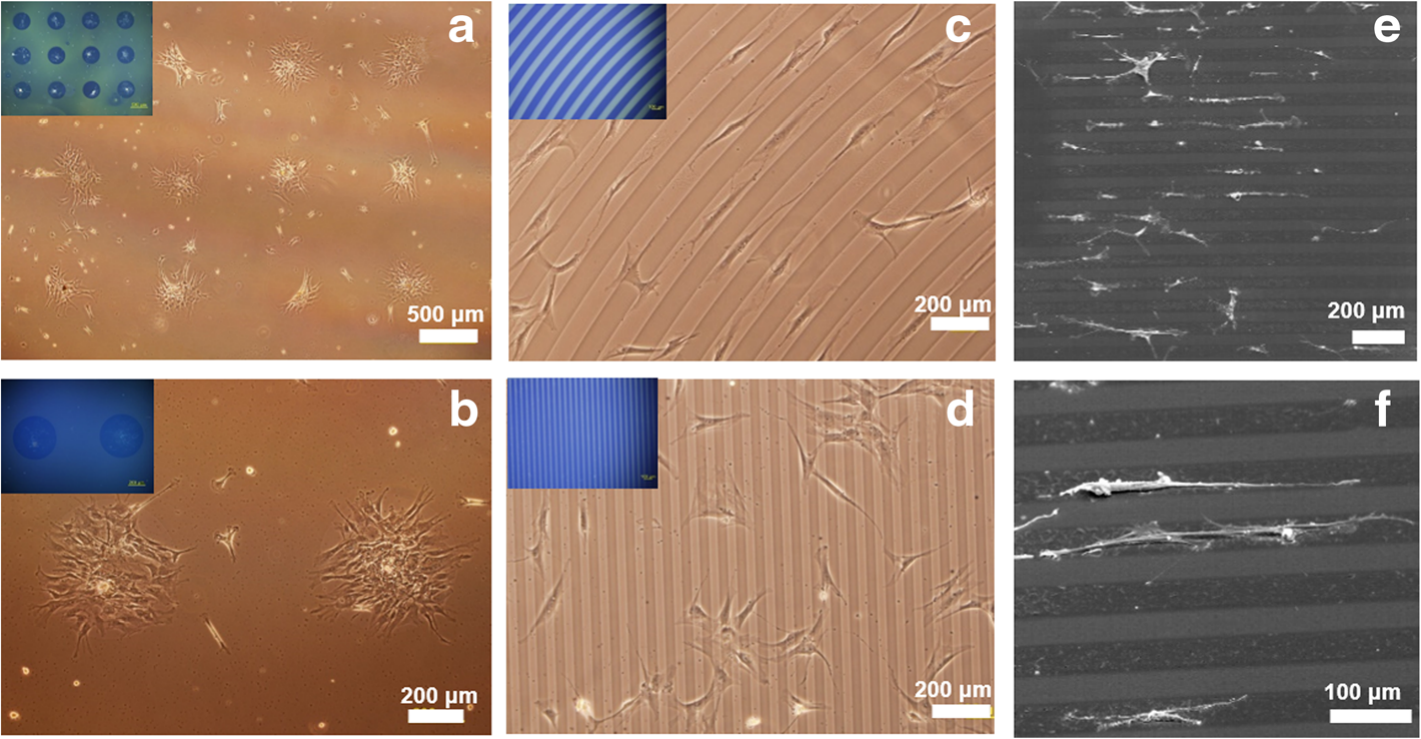
Optical microscopic images of MSCs cultured on (**a** and **b**) 500 μm dimeter circle, (**c**) 50 μm wide curved, (**d**) 20 μm wide line patterns of PMAn films. Inset: The fluorescent microscope images of the same patterns. (**e** and **f**) Scanning electron microscope images of MSCs pattern on 50 μm wide line patterns of PMAn film

It has been known that MSCs were selectively attached on a hydrophilic surface [23, 32, 33]. Thus, micro patterned PMAn substrate via photo-oxidation was expected for the formation of MSC patterning. MSCs were seeded onto the fluorescent patterned surface of PMAn in a culture medium and allowed to attach for overnight. By comparison with the random attachment of MSCs on tissue culture polystyrene, Fig. 6a-c clearly show that MSCs were preferentially attached on the UV exposed region of circle pattern with 500 um diameter and were aligned in the direction of the line pattern with 50 un width. This is probably because UV exposed regions became hydrophilic surface, promoting the attachment of MSCs. Interestingly, the pattern formation of MSCs was dependent on the pattern size of PMAn. On the line pattern with < 20 μm width, MSCs didn’t show selective adhesion on UV exposed region and were randomly attached on the whole pattern surface (Fig. 6d). This may be ascribed to the size of MSCs, which are generally larger than 20 μm. Figure 6e and f show the SEM images of MSCs attached on UV exposed regions and aligned in the direction of the line pattern of the PMAn. Overall, the proliferation assay, optical microscopic and SEM images clearly support that the proliferation as well as the attachment of MSCs was much more favorable on the hydrophilic UV exposed regions of PMAn than UV unexposed regions of PMAn.

## Conclusions

We have shown simple cell patterning method that doesn’t require labor-intensive preparation for cell patterning. The UV exposure to PMAn film leads to fluorescent quenching as well as the change in surface wettability into hydrophilic surface due to direct photo-reactions of anthracene units. In contrast to UV unexposed regions of PMAn film (PMAn-UV), UV exposed regions of PMAn film (PMAn + UV) showed high proliferation rate and selective attachment of MSCs. We envision that this simple cell pattern method may be valuable for designing cellular platform for drug screening, diagnostics, and cellular/tissue engineering.

## Abbreviations

AFM, atomic force microscopy; Ar, argon; FBS, Fetal Bovine Serum; GPC, gel permeation chromatography; MSCs, mesenchymal stem cells; PBS, Phosphate-Buffered Saline; PEG, poly (ethylene glycol); PMAn, anthracene containing polymers; PMAn + UV, UV exposed PMAn film; PMAn-UV, UV unexposed PMAn film; PPV, *p*-phenylene vinylene; SAMs, self-assembled monolayers; TCPS, tissue culture polystyrene; α-MEM, α-Minimum Essential Medium;
