# Erratum to: Direct photo-patterning on anthracene containing polymer for guiding stem cell adhesion

**DOI:** 10.1186/s40824-016-0075-1

**Published:** 2016-09-02

**Authors:** Jungmok You, June Seok Heo, Hyun Ok Kim, Eunkyoung Kim

**Affiliations:** 1 Department of Plant & Environmental New Resources, Kyung Hee University, 1732 Deogyeong-daero, Giheung-gu, Yongin-si, Gyeonggi-do 446-701 South Korea; 2 Cell Therapy Center, Severance Hospital, Yonsei University College of Medicine, Seoul, South Korea; 3 Department of Laboratory Medicine, Yonsei University College of Medicine, Seoul, South Korea; 4 Department of Chemical and Biomolecular Engineering, Yonsei University, 262 Seongsanno, Seodaemun-gu, Seoul 120-749 South Korea

## Erratum

*n.b. The associated online manuscript has now been updated with this correction accordingly.*

- The original version of the manuscript [1] displayed the lead author’s name as “Eunkyoumg Kim”, whereas it should have read “Eunkyoung Kim”.
